# Multiplexed targeted mass spectrometry assays for prostate cancer-associated urinary proteins

**DOI:** 10.18632/oncotarget.21710

**Published:** 2017-10-09

**Authors:** Tujin Shi, Sue-Ing Quek, Yuqian Gao, Carrie D. Nicora, Song Nie, Thomas L. Fillmore, Tao Liu, Karin D. Rodland, Richard D. Smith, Robin J. Leach, Ian M. Thompson, Elizabeth A. Vitello, William J. Ellis, Alvin Y. Liu, Wei-Jun Qian

**Affiliations:** ^1^ Biological Sciences Division, Pacific Northwest National Laboratory, Richland, WA, USA; ^2^ Department of Urology, University of Washington, Seattle, WA, USA; ^3^ Institute for Stem Cell and Regenerative Medicine, University of Washington, Seattle, WA, USA; ^4^ Environmental Molecular Sciences Laboratory, Pacific Northwest National Laboratory, Richland, WA, USA; ^5^ Department of Urology and the Cancer Therapy and Research Center, University of Texas Health Science Center at San Antonio, San Antonio, TX, USA; ^6^ Present address: Singapore Polytechnic, Center for Biomedical and Life Sciences T11A-412 (level 4), Singapore

**Keywords:** secreted protein biomarkers, prostate cancer detection, prostate cancer, targeted mass spectrometry, selected reaction monitoring

## Abstract

Biomarkers for effective early diagnosis and prognosis of prostate cancer are still lacking. Multiplexed assays for cancer-associated proteins could be useful for identifying biomarkers for cancer detection and stratification. Herein, we report the development of sensitive targeted mass spectrometry assays for simultaneous quantification of 10 prostate cancer-associated proteins in urine. The diagnostic utility of these markers was evaluated with an initial cohort of 20 clinical urine samples. Individual marker concentration was normalized against the measured urinary prostate-specific antigen level as a reference of prostate-specific secretion. The areas under the receiver-operating characteristic curves for the 10 proteins ranged from 0.75 for CXL14 to 0.87 for CEAM5. Furthermore, MMP9 level was found to be significantly higher in patients with high Gleason scores, suggesting a potential of MMP9 as a marker for risk level assessment. Taken together, our work illustrated the feasibility of accurate multiplexed measurements of low-abundance cancer-associated proteins in urine and provided a viable path forward for preclinical verification of candidate biomarkers for prostate cancer.

## INTRODUCTION

Prostate cancer is the most common solid tumor in men and the second leading cause of male cancer-related deaths in the US. Over-diagnosis and over-treatment of prostate cancer have become major concerns for disease management ever since the introduction of serum prostate-specific antigen (sPSA) screening [1, 2]. There is still a significant need to develop informative biomarkers for effective non-invasive detection of high risk prostate cancer, the ones that need to be treated, from the many low risk non-life threatening cancer cases.

Human urine is an ideal clinical specimen for testing prostate cancer biomarkers since prostatic secretion passes into the urine. Currently, one prostate cancer urine test measures a cancer-specific non-coding transcript PCA3 released from prostate cancer cells [3]. In a cohort of >500 patients with serum PSA between 3 and 15 ng/mL, the area under the receiver-operating characteristic curve (AUC) was 0.66 with a sensitivity of 65% and a specificity of 66% [4]. As a prognostic marker, PCA3 showed no significant link to Gleason score, tumor volume, and cancer stage in a cohort of 70 cases [5], though a link to tumor volume and surgical margin was reported in another study [6]. PCA3 is a low abundance transcript, and an “attentive” digital rectal exam (DRE) by an experienced urologist is required to enhance the PCA3 signal [7]. Since most current clinical tests are based on protein analytes, there is an interest in identifying better protein biomarkers for prostate cancer. Moreover, proteins are more stable than RNA, which requires the addition of a preservative to the urine sample and immediate processing.

We have previously identified a set of prostate cancer-associated secreted protein markers by cell-type transcriptomics [8, 9] for quantification in urine. Assay developments for measuring single secreted protein markers in voided urine have been reported [8–12]. For example, AGR2 (anterior gradient 2) is produced in relatively high abundance by cancer epithelial cells [9]. Compared with benign tissue, AGR2 is highly expressed in tumors at the mRNA and protein levels [10, 13]. A sandwich ELISA and a highly sensitive targeted mass spectrometric approach termed PRISM (high-pressure, high-resolution separation with intelligent selection and multiplexing) coupled with selected reaction monitoring (SRM) were used to measure AGR2 in human urine at pg/mL levels [11]. We demonstrated that the amounts of urinary AGR2 measured by both ELISA and PRISM-SRM in the same samples were concordant with *R^2^* = 0.91. Our initial cohort study indicated that urinary AGR2 was able to differentiate prostate cancer from non-cancer urine with an AUC = 0.75 [11].

Herein, we report multiplexed measurements of 12 cancer-associated proteins in urine by targeted mass spectrometry (MS) and the potential utility of these markers for prostate cancer detection. SRM-based targeted MS has proven to be a reliable technology for accurate quantification of target proteins due to its high reproducibility, multiplexing, and specificity whereas antibodies can sometimes show unexpected cross reactivity [14, 15]. A major limitation of typical liquid chromatography (LC)-SRM analysis is the insufficient sensitivity to detect low-abundance proteins in body fluids (e.g., <1 ng/mL in blood plasma/serum), encountered as in early detection [14]. We recently introduced two highly sensitive complementary targeted proteomics approaches: long gradient (LG)-SRM [16] and PRISM-SRM [17, 18] for reliable detection and quantification of low-abundance proteins in body fluids and human tissues. LG-SRM and PRISM-SRM were demonstrated to provide ≥10-fold and ≥200-fold higher sensitivity, respectively, when compared to standard LC-SRM. To enable multiplexed quantification of prostate cancer associated protein markers in urine, we have developed sensitive SRM assays for direct detection of these markers in voided urine without entailing DRE. The multiplexed SRM assays provide a means for verifying the performance of individual markers or multi-marker panel for prostate cancer detection. Once promising markers are identified and verified in initial cohort studies, antibody-based ELISA assays can be developed for high-throughput clinical applications.

## RESULTS

### Tumor-associated secreted proteins in human urine

Through comparison of cell type-specific transcriptomes, genes showing elevated tumor expression and encoding secreted/extracellular proteins were identified from both the epithelial and stromal compartments. Furthermore, gene expression analysis indicated that many showed differential expression among tumors of different Gleason scores. The epithelial derived marker candidates included AGR2, AGR3, CRISP3, CEAM5, CEAM6, CCL3, CCL4, IL24, MMP9; the stromal derived candidates included CXL14, CD90, IL24, MMP9, POSTN, SFRP4, and WISP1. In the *UrinePA* (peptide atlas, http://www.peptideatlas.org) archive of proteome datasets, the “observed” (in brackets) qualifier was used to indicate protein abundance. Of the marker candidates, CRISP3 (65), CEAM5 (21), CEAM6 (5), CD90/*THY1* (261), MMP9 (115), SFRP4 (17) were listed (Supplementary Table 1). Those that were not detected in healthy donors could be either below the limit of detection or likely specific for disease (e.g., prostate cancer).

### Multiplexed SRM assays for prostate cancer protein markers

To develop targeted SRM assays for individual proteins, selection of the most suitable surrogate peptides for each protein was critical for precise quantification of target proteins in patient specimens. The initial selected surrogate peptides for each protein marker are listed in Supplementary Table 2. The peptide selection follows several main criteria: a) sequences being unique to their corresponding proteins; b) peptides having high MS response and minimal matrix interference in LC-SRM signals; c) generally no known modifications or mutations within the selected peptide sequences.

**Figure 1:**
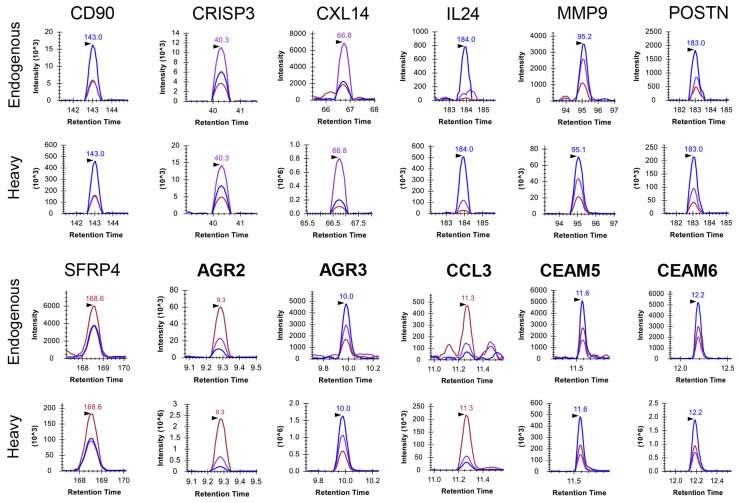
Extracted ion chromatograms (XICs) of detected proteins in a single urine sample, P07-031C Seven proteins (CD90, CRISP3, CXL14, IL24, MMP9, POSTN, SFRP4) were detected by LG-SRM, and the other five (AGR2, AGR3, CCL3, CEAM5, CEAM6) in extremely low abundance were detected by PRISM-SRM. Three transitions (blue, chestnut, and purple curves) for one surrogate peptide of each protein were monitored. The surrogate peptides being monitored are: CD90 (*THY1*): VLYLSAFTSK, CRISP3: WANQC_cam_NYR, CXL14: MVIITTK, IL24: LWEAFWAVK, MMP9: AVIDDAFAR, POSTN: AAAITSDILEALGR, SFRP4: GVC_cam_ISPEAIVTDLPEDVK, AGR2: LPQTLSR, AGR3: LYTYEPR, CCL3: QVC_cam_ADPSEEWVQK, CEAM5: SDLVNEEATGQFR, CEAM6: SDPVTLNVLYGPDGPTISPSK.

For PSA, IVGGWECEK and LSEPAELTDAVK were demonstrated to be the most effective [17, 19]. For the others, a pooled prostate cancer patient urine sample was used to configure the final SRM assays with evaluation of matrix interference, endogenous peptide detectability and peptide SRM response. LG-SRM was used first to measure all candidates simultaneously due to its moderate sensitivity (≥ 10-fold higher than LC-SRM) and higher multiplexing capability (~3 times higher than LC-SRM) [16]. PSA, CD90, CRISP3, CXL14, IL24, MMP9, POSTN, and SFRP4 were confidently detected and quantified by at least one surrogate peptide (Figure 1 and Table 1). More sensitive PRISM-SRM (≥20-fold higher in sensitivity than LG-SRM [17]) was then used to measure the remainder. AGR2, AGR3, CCL3, CEAM5, and CEAM6 were reliably detected and quantified except CCL4 and WISP1 (Figure 1). The reproducibility of LG-SRM and PRISM-SRM based assays for measurements in biofluids such as urine and serum was well validated in our previous reports, which typically had a coefficient of variance (CV) <10% [16, 17, 20]. With a combined LG-SRM and PRISM-SRM, SRM assays were established for each of the detectable peptides: three best transitions without matrix interference and the best transition for quantitation (Table 1). We note that two peptides, LYTYEPR for AGR3 and MVIITTK for CXL14, may not serve as good surrogates for protein quantification because of the reported phosphorylation sites as well as the potential oxidation on the methionine residue for MVIITTK.

**Table 1:** Prostate cancer-associated secreted proteins and their surrogate peptides

Protein	Accession number	Best surrogated peptide*^a^*	SRM transitions			
			Q1		Q3	
AGR2	O95994	LPQTLSR	407.7	604.3	476.3	351.2
AGR3	Q8TD06	LYTYEPR	471.2	665.3	272.2	277.2
CCL3	P10147	QVCADPSEEWVQK*^b^*	788.4	1188.6	1117.5	1002.5
CEAM5	P06731	INGIPQQHTQVLFIAK	603.0	847.5	761.9	705.4
		SDLVNEEATGQFR	733.3	1051.5	937.4	679.4
		CETQNPVSAR*^b^*	581.3	872.5	643.4	529.3
CEAM6	P40199	EVLLLAHNLPQNR	506.3	741.4	514.3	531.8
		SDPVTLNVLYGPDGPTISPSK	1079.1	1055.5	998.5	331.2
CRISP3	P54108	WANQCNYR*^b^*	556.2	925.4	854.4	612.3
		YEDLYSNCK*^b^*	596.3	899.4	784.4	671.3
CXL14	O95715	MVIITTK	403.2	674.4	575.4	462.3
		WYNAWNEK	555.8	761.4	647.3	576.3
IL24	Q13007	LWEAFWAVK	575.3	850.4	721.4	650.4
MMP9	P14780	AVIDDAFAR	489.3	807.4	694.3	579.4
		FQTFEGDLK	542.8	809.4	708.4	561.3
		LGLGADVAQVTGALR	720.9	914.5	815.5	744.4
		SLGPALLLLQK	576.9	952.6	727.5	614.4
POSTN	Q15063	AAAITSDILEALGR	700.9	1074.6	973.5	771.5
SFRP4	Q6FHJ7	GVCISPEAIVTDLPEDVK*^b^*	971.5	1425.7	916.5	587.3
CD90	P04216	VLYLSAFTSK	564.8	916.5	753.4	640.3
		VTSLTACLVDQSLR*^b^*	521.6	830.5	717.4	618.3
		HVLFGTVGVPEHTYR	571.3	958.5	802.4	576.3

*^a^*These surrogate peptides were confidently detected in the pooled urine sample.

*^b^*Cysteine was synthesized as carbamidomethyl cysteine.

From the assay results, the 12 detected markers were grouped into 7 moderate-to-low abundance proteins for LG-SRM and 5 low abundance proteins for PRISM-SRM. CCL4 and WISP1 were excluded from further testing. The SRM assays were then applied for marker quantification in a cohort of 14 cancer (pre-op) and 6 non-cancer (healthy control) urine collected at the University of Washington (UW), and a cohort of post-op urine collected at the University of Texas Health Science Center at San Antonio (UTHSCSA) for urinary PSA contribution by the prostate. Among the 12 proteins, 10 proteins can be reliably detected and quantified across the 20 urine subjects with at least one surrogate peptide, except CCL3 and POSTN.

### Concordance between multiple surrogate peptides from the same protein

**Figure 2:**
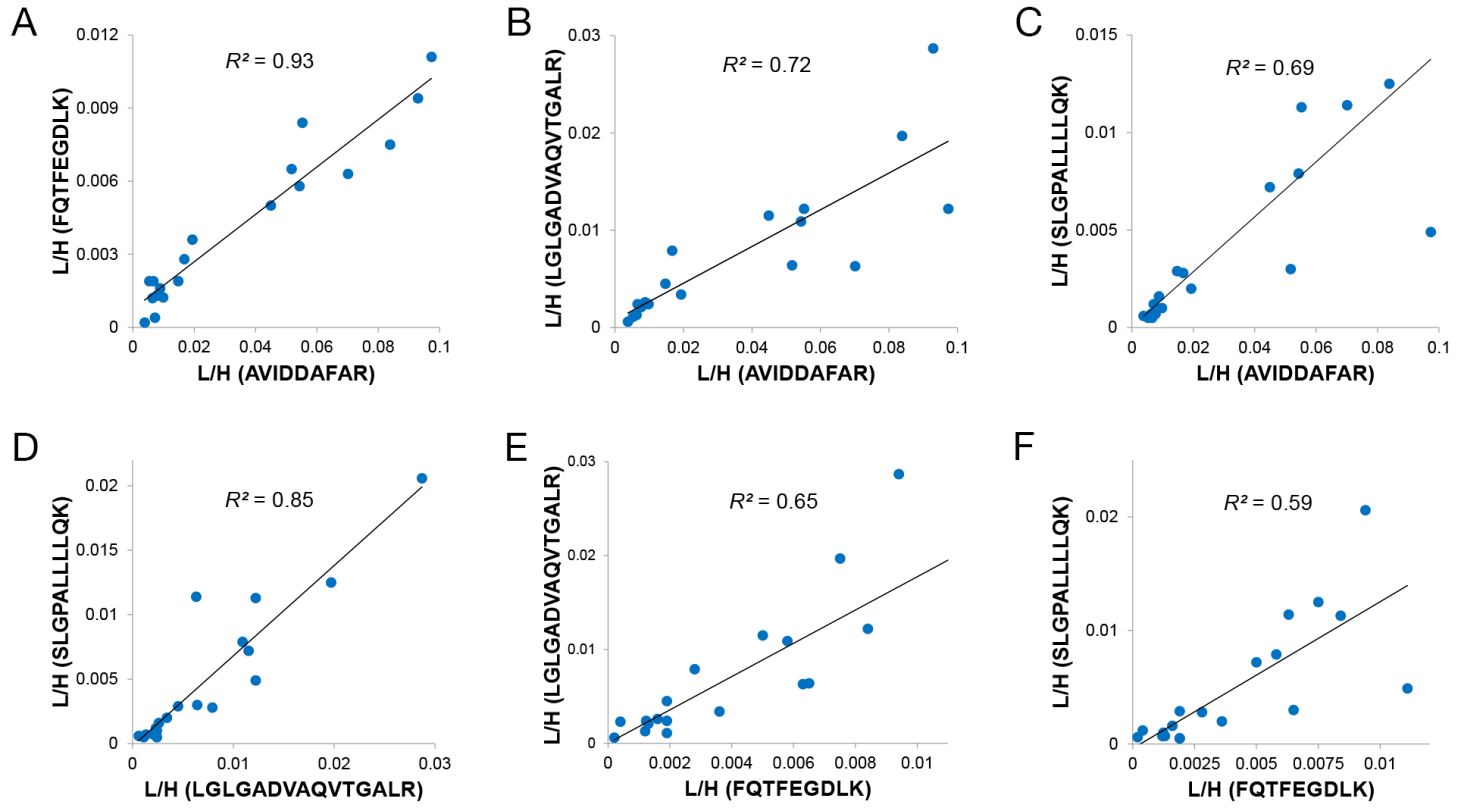
Correlation plot between any two MMP9 surrogate peptides in 20 urine samples **(A)** Relative abundance correlation between FQTFEGDLK (*y*-axis) and AVIDDAFAR (*x*-axis); **(B)** Relative abundance correlation between LGLGADVAQVTGALR (*y*-axis) and AVIDDAFAR (*x*-axis); **(C)** Relative abundance correlation between SLGPALLLLQK (*y*-axis) and AVIDDAFAR (*x*-axis); **(D)** Relative abundance correlation between SLGPALLLLQK (*y*-axis) and LGLGADVAQVTGALR (*x*-axis); **(E)** Relative abundance correlation between LGLGADVAQVTGALR (*y*-axis) and FQTFEGDLK (*x*-axis); **(F)** Relative abundance correlation between SLGPALLLLQK (*y*-axis) and FQTFEGDLK (*x*-axis). L/H = the ratio of SRM signal from endogenous peptide over heavy-labeled internal standard. *R^2^* values range from 0.59 to 0.93.

Since we selected multiple surrogate peptides for quantification of a specific protein in urine, we evaluated the agreement between these peptides from the same protein. Conceptually, when no posttranslational modifications or undocumented amino acid changes exist in the surrogate peptides, their measured concentrations should have a high degree of correlation across all samples because the surrogate peptide level was stoichiometric to that of their cognate protein [21]. With any peptide sequence modifications, the level of the unmodified surrogate peptides would be lower, affecting accurate measurement of their corresponding proteins. Given the possibility of unknown sequence modifications, each surrogate peptide could potentially represent a distinctive signature with diagnostic value [22]. To evaluate the quantification accuracy, correlation analysis of the L/H ratios between the surrogate peptides from the same protein was carried out. For example, MMP9 was represented by four quantifiable surrogate peptides, and the Pearson correlation coefficients ranged from 0.59 for FQTFEGDLK and SLGPALLLLQK to 0.93 for AVIDDAFAR and FQTFEGDLK, which suggested that multiple MMP9 isoforms could exist in these clinical urine samples (Figure 2). For CD90, low correlation coefficients between VTSLTACLVDQSLR and two other peptides were obtained, whereas a good correlation, *R^2^* = 0.72, was found for the other two peptides (Supplementary Figure 1). This suggested the presence of unknown modifications in VTSLTACLVDQSLR in several urine samples, making this peptide unsuitable for accurate measurement of CD90.

### The origin of urinary PSA

To assess whether urinary PSA is exclusively originating from the prostate, LC-SRM was used to measure its concentrations in 7 urine samples from men after radical prostatectomy (i.e., the entire prostate being removed) and the cohort of 20 urine samples before radical prostatectomy (Supplementary Table 3). The measured PSA levels ranged from 0.02 ng/100 μg to 2.95 ng/100 μg of total protein with an average value of 0.98 ng/100 μg (median 0.41 ng/100 μg, Supplementary Table 4). When compared with the PSA levels in the others with an average value of 110.89 ng/100 μg of total urinary protein (median 28.68 ng/100 μg), the PSA percentage in the post-op urine was ~1% (median ~1.5%, Supplementary Table 4). Thus, our data showed that urinary PSA was secreted exclusively from the prostate, and the contribution from other sources in the urinary system was negligible.

### Initial assessment of marker utility in a pilot cohort

In SRM measurements, the L/H peak area ratios were proportional to the concentrations of their cognate protein, which were expressed as ng/100μg of total urinary protein because of the same peptide concentration with the same amount of spiked-in heavy internal standards (see Supplementary Methods). Thus, the L/H ratio could be regarded as the adjusted concentration of the target protein in urine (against the total amount of urinary proteins [11], Supplementary Table 5). This adjustment accounted for a substantial degree of variations in urinary protein concentration among donors, and donations from the same donor. For most surrogate peptides measured, the cancer urine showed higher median L/H values than non-cancer urine; while for several others (CRISP3, CXL14, IL24 and SFRP4), a lower or equal median L/H value in cancer *vs*. non-cancer was found. A Mann-Whitney U test of the surrogate peptide L/H ratios revealed no significant difference between cancer and non-cancer for all the markers (Table 2).

**Table 2:** Performance of surrogate peptide markers derived from 10 prostate cancer-associated secreted proteins in 20 urine samples (14 cancer and 6 non-cancer samples)

Protein	Peptide	(L/H)_peptide marker_		(L/H)_peptide marker_/(L/H)_PSA_			
		*P* value*^a^*	AUC	*P* value*^a^*	AUC	Sensitivity*^b^*	Specificity*^b^*
AGR2	LPQTLSR	0.773	0.45	0.063	0.77	0.93	0.67
AGR3	LYTYEPR	0.283	0.66	0.019	0.85	0.79	1
CEAM5	SDLVNEEATGQFR	0.322	0.65	0.012	0.87	0.71	1
CEAM6	EVLLLAHNLPQNR	0.246	0.67	0.029	0.82	0.79	0.83
CRISP3	WANQCNYR*^c^*	0.386	0.63	0.035	0.86	0.86	0.83
CRISP3	YEDLYSNCK*^c^*	0.433	0.38	0.035	0.81	0.64	1
CD90	VLYLSAFTSK	0.174	0.70	0.015	0.86	0.86	0.83
CD90	VTSLTACLVDQSLR*^c^*	0.967	0.45	0.063	0.77	0.64	1
CD90	HVLFGTVGVPEHTYR	0.650	0.57	0.012	0.87	0.86	0.83
CXL14	MVIITTK	0.836	0.46	0.091	0.75	0.79	0.83
IL24	LWEAFWAVK	0.479	0.61	0.015	0.86	0.71	1
MMP9	AVIDDAFAR	1	0.50	0.029	0.82	0.93	0.67
MMP9	FQTFEGDLK	0.710	0.56	0.015	0.86	0.93	0.67
MMP9	LGLGADVAQVTGALR	0.869	0.47	0.015	0.86	0.86	0.83
MMP9	SLGPALLLLQK	1	0.49	0.015	0.86	0.85	0.83
SFRP4	GVCISPEAIVTDLPEDVK*^c^*	0.592	0.42	0.023	0.83	0.64	1

*^a^P* values were obtained from the Mann-Whitney U test.

*^b^*These are the sensitivity and specificity at the optimal cutoff point (i.e., the best sum of sensitivity and specificity).

*^c^*Cysteine was synthesized as carbamidomethyl cysteine.

Since prostate cancer associated proteins are mostly secreted from the prostate tissue, we considered a “normalization” strategy against a baseline level of prostate specific secretion. For this purpose, we adapted the strategy to normalize all marker concentrations against urinary PSA concentration. We chose urinary PSA level as a reference value of prostate specific secretion because our data showed that urinary PSA was exclusively secreted from the prostate gland. Similar normalization strategy was applied in the urine PCA3 assay where the marker score was generated by normalization of the PCA3 transcript levels to those of PSA transcript [23].

**Figure 3:**
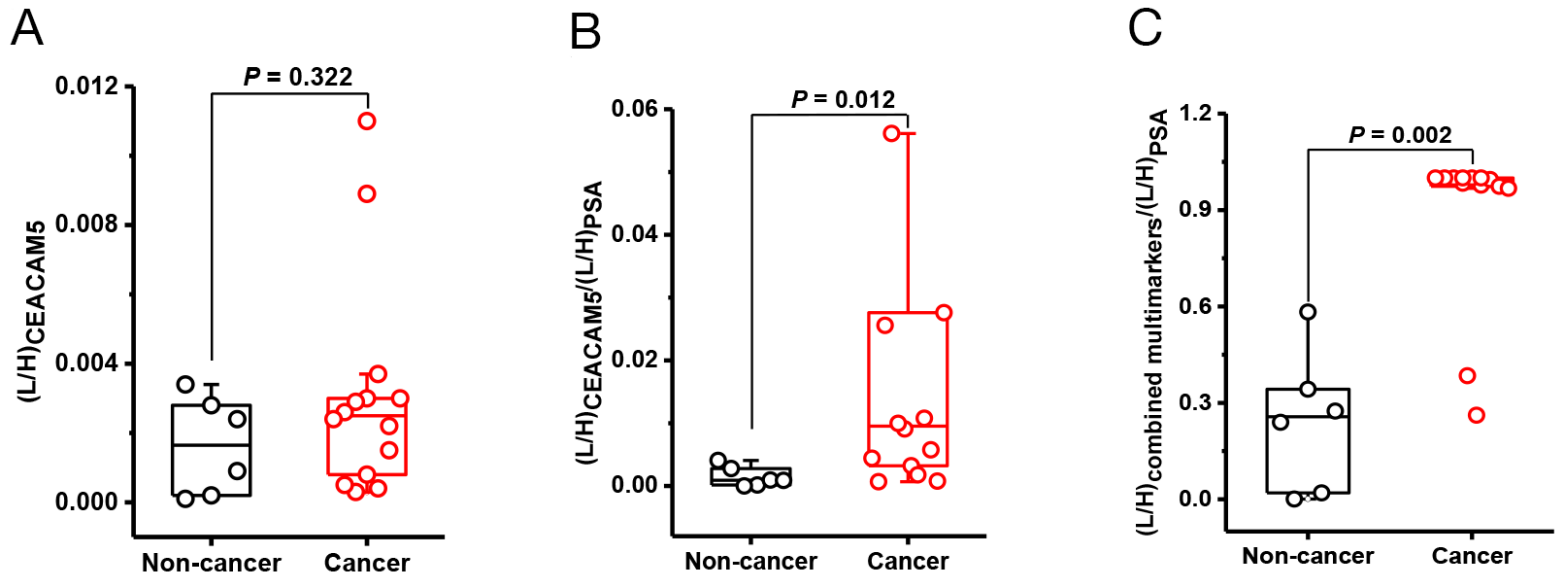
Urine protein biomarkers for prostate cancer **(A)** CEAM5 relative abundance between non-cancer (n = 6) and cancer urine (n = 14), *P* = 0.322; **(B)** CEAM5/PSA concentration ratios between non-cancer and cancer, *P* = 0.012; **(C)** Significant differentiation between non-cancer and cancer, *P* = 0.0034, with the best peptide combination. The relative abundance of CEAM5 and PSA was derived from their surrogate peptides, SDLVNEEATGQFR and IVGGWEC_cam_EK, respectively. The best peptide combination: LPQTLSR/AGR2, LYTYEPR/AGR3, SDLVNEEATGQFR/CEAM5, VTSLTACLVDQSLR/CD90, and GVCISPEAIVTDLPEDVK/SFRP4.

The protein marker/PSA concentration ratios were obtained by dividing the L/H peak area ratio of surrogate marker peptides by that of PSA peptide IVGGWEC_cam_EK (Supplementary Table 6). After PSA normalization, a significant difference between the cancer and non-cancer urine was observed for the marker peptides [except for LPQTLSR of AGR2, VTSLTACLVDQSLR of CD90 and MVIITTK of CXL14] with *P* = 0.015-0.035 (Table 2 and Figure 3). ROC analysis with 95% confidence intervals showed that the peptides with *P* < 0.05 produced AUC values >0.80, while for the three peptides with *P* > 0.05 the AUC values produced were <0.80 (Table 2). These analyses indicated some of the biomarkers have potential utilities in the detection of prostate cancer.

Furthermore, our data show that peptides from the same protein with a good correlation produced similar AUC values. For example, the MMP9 peptides - AVIDDAFAR, FQTFEGDLK, LGLGADVAQVTGALR, SLGPALLLLQK - produced values of 0.82, 0.86, 0.86, and 0.86, respectively, as did the two well-correlated CD90 surrogate peptides: VLYLSAFTSK (0.86), HVLFGTVGVPEHTYR (0.87). VTSLTACLVDQSLR without significant correlations produced an AUC value of 0.77 (Table 2). The data suggests that the concentration of a given protein can be accurately quantified based on multiple well-correlated surrogate peptides. Multi-marker performance was also assessed by using multivariate analysis of various peptide combinations from different proteins (Figure 3 and Supplementary Table 7) and the combination of all surrogate peptides from the same protein (Supplementary Figure 3 and Supplementary Table 8). The best combination was LPQTLSR/AGR2, LYTYEPR/AGR3, SDLVNEEATGQFR/CEAM5, VTSLTACLVDQSLR/CD90, and GVCISPEAIVTDLPEDVK/SFRP4 with *P* = 0.002 and AUC = 0.95.

### Detection of clinically significant cancer by secreted protein markers

**Figure 4:**
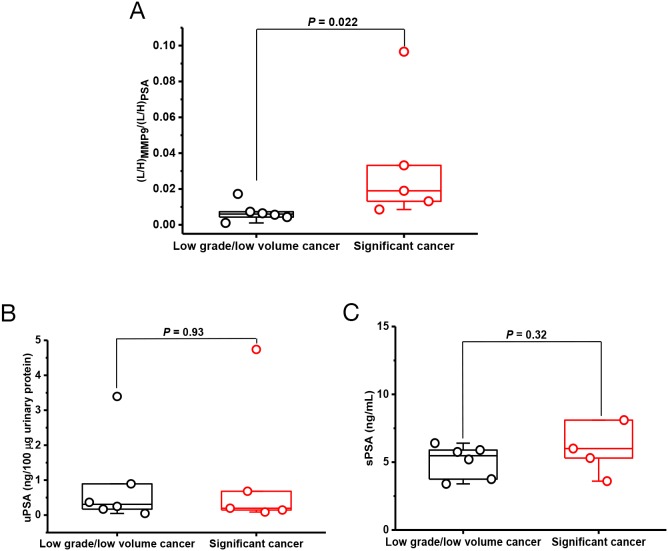
Stratification of prostate cancer based on tumor volume and Gleason score **(A)** The relative abundance ratios of FQTFEGDLK/MMP9 over IVGGWEC_cam_EK/PSA between low volume/low grade cancer (n = 6) and significant cancer (n = 5), *P* = 0.022; **(B)** Urinary PSA concentrations (uPSA) between low volume/low grade cancer and significant cancer, *P* = 0.93; **(C)** Serum PSA concentrations (sPSA) between low volume/low grade cancer and significant cancer, *P* = 0.32.

Next, we test the potential to differentiate high-risk cancer from low grade cancer. The prostate cancer cohort was grouped into either low volume/low grade (Gleason score ≤6 and tumor volume ≤ 0.5 cc [24]) or clinically significant (not meeting the above criteria for low volume/low grade disease, Supplementary Tables 9 and 10). The significance for most markers in identifying the high-risk cancers was not apparent except with MMP9 (Supplementary Table 10). The two surrogate peptides, FQTFEGDLK and LGLGADVAQVTGALR, produced *P* value of 0.022 in comparing low volume/low grade cancer and significant cancer (Figure 4 and Supplementary Table 10). The observation suggested an association between MMP9 and high grade/volume in this patient cohort. This result was also supported by cell-type transcriptomics data. Array signal intensity value for MMP9 in Gleason 4 cancer cells was 3004.10, ~12-fold higher than that of 238.41 in Gleason 3 cancer cells. (Supplementary Figure 2). For comparison, urine PSA and serum PSA showed no significance between the two cancer groups (Figure 4).

## DISCUSSION

To date, disease detection relies mostly on single markers. The concept of multi-marker panel has the potential for more specific disease diagnosis and prognosis. Our data demonstrated the feasibility and promising aspects of multiplexed targeted MS assays for low-abundance prostate cancer-associated proteins in voided urine. The development for such assays is generally rapid in identifying the right surrogate peptides and implementation when compared with immunoassays that require the time-consuming generation of high quality monoclonal antibodies and their validation testing. With continuous advancement in measurement sensitivity, (e.g., LG-SRM [16] or PRISM-SRM [17]), SRM assays are feasible for sensitive measurement of low-abundance protein biomarkers in tissues [25, 26] and human body fluids [11, 27], as well as for facilitating the transition of biomarkers to large-scale clinical validation trials.

One important feature for targeted MS assays is that multiple surrogate peptides can be selected for a given protein. Each surrogate peptide from a given protein can serve as a unique marker since it may contain unique PTM or other sequence modifications. Without such modifications, the abundances for any two or more surrogate peptides from the same protein should correlate well across many samples. In studies involving human cell lines, most surrogate peptides (453/466) showed a high correlation coefficient (*R^2^* > 0.8) [21]. However, many surrogate peptides used in our urine analysis were found to have moderate correlation coefficients (median *R^2^* = 0.70) with data point deviations. This observation suggests that the target proteins in patient urine samples are more varied than those in single cell lines most likely due to allelic differences or isoforms. Therefore, multiple surrogate peptides per protein need to be tested in assay development and the individual peptide signatures may provide additional values for disease detection.

One challenge to urinary marker quantification is the large variation of urine protein concentration, and normalization strategies are often necessary. In our study, we observed that PSA as a prostate-specific secretory marker serves as an effective reference for normalization of other prostate cancer-associated proteins. Without PSA normalization, the performance for most markers was poor because of the multiple tissue sources of the urine proteome. Our assumption is that the main source of our panel of prostate cancer-associated proteins is from prostate cancer cells. By normalization against urinary PSA, a marker reflecting the total prostate cells, the marker performance was significantly improved. The significantly higher concentrations of urinary PSA found in some non-cancer samples could be due to donors with an enlarged prostate from benign hyperplasia. For example, prostate cancer patients with prostate volume of 35 cm^3^ (n = 29) and benign prostatic hyperplasia patients prostate volume of 45 cm^3^ (n = 35) were measured to have median urinary PSA levels of 52.6 ng/mL and 123.2 ng/mL, respectively [28].

The eventual goal of developing an informative panel of biomarkers is to reduce the need for prostate biopsy, an invasive, expensive, and potentially morbid procedure with up to a 4% risk of sepsis [29]. One could envision that prostate cancer diagnosis would involve the use of a relatively small number of markers as a tool for cancer detection, perhaps as a “reflex test” after PSA testing when the patient has an abnormal serum PSA. Notably, if the multi-marker panel is negative, no biopsy would be necessary especially when the negative predictive value is sufficiently high. Furthermore, our marker panel (e.g., MMP9) could have the potential utility in distinguishing low grade/low volume cancer from significant cancer. Therefore, by effectively integrating multi-marker measurement results, there is a greater possibility for detection of significant cancer with fewer biopsies performed in patients without cancer.

In conclusion, through comparison of cell type-specific transcriptomes, 14 cancer-associated secreted proteins were identified as candidate biomarkers. Sensitive multiplexed targeted MS assays were developed for reliable quantification of 10 secreted proteins (including previously reported AGR2) in human urine. All markers can be reproducibly detected and quantified in all the urine samples with at least one surrogate peptide. Most of the markers appear to be promising in prostate cancer detection in a pilot cohort study with initial AUC ranging from 0.75 to 0.86. Further studies with additional large sample cohorts to fully validate the performance of these markers are warranted. Our sensitive targeted SRM assays should also facilitate biomarker analysis of other cancers, especially for markers like secreted AGR2 that are widely present in many tumor types.

## MATERIALS AND METHODS

### Urine collection

The use of human urine samples was approved by the Institutional Review Boards of the University of Washington (UW), Pacific Northwest National Laboratory (PNNL), and the University of Texas Health Science Center at San Antonio (UTHSCSA). Samples from consented donors were anonymized before given to the researchers. Suffix N added to the sample codes denoted non-cancer, and suffix C denoted cancer from pre-op patients. Post-op urine was collected after surgical resection of the prostate.

### Chemical reagents

Urea, dithiothreitol (DTT), iodoacetamide, ammonium formate, trifluoroacetic acid (TFA) and formic acid were purchased from Sigma (St. Louis, MO). The synthetic peptides labeled with ^13^C/^15^N on C-terminal lysine and arginine residues were from Thermo Scientific (San Jose, CA). The heavy peptides for PSA protein were estimated to be of >95% purity by HPLC.

### Urine processing and protein digestion

Collected voided urine samples were processed within 2 h (to isolate RNA as well). The samples were centrifuged at 1,200 rpm for 5 min and the supernatant was stored at -80°C. Fifteen-90 mL of urine were desalted and concentrated using Amicon^®^ Ultra-15 (3 kDa molecular weight cut-off, Millipore, Billerica, MA) [12]. Protein concentrations were determined by the BCA assay (Pierce, Rockford, IL). Concentrated urinary proteins from each sample, ranging from 200 to 300 μg, were denatured and reduced with 8 M urea and 10 mM DTT in 50 mM NH_4_HCO_3_, pH 8.0 for 1 h at 37°C. Protein cysteine residues were alkylated with 40 mM iodoacetamide for 1 h at room temperature in the dark. The resulting sample was diluted 6-fold with 50 mM NH_4_HCO_3_, pH 8.0, and digested by sequencing-grade modified porcine trypsin (Promega, Madison, WI) at 1:50 trypsin:protein (w/w) overnight at 37°C. The resulting digest was desalted by using 1 mL-SPE C18 column (Supelco, Bellefonte, PA) as described previously [11]. The final tryptic peptide concentration was determined by BCA. The peptide sample was diluted to 0.5 μg/μL with 0.1% formic acid in water, and crude heavy isotope-labeled synthetic peptides of protein markers were spiked in at an equimolar concentration of 10 fmol/μL; 10 fmol/μL of pure heavy peptide IVGGWEC_cam_EK (C_cam_: cysteine residue synthesized as carbamidomethyl cysteine) and 1 fmol/μL of pure heavy peptide LSEPAELTDAVK of PSA.

### Database query

The human urine proteome databases archived in PeptideAtlas (http://www.peptideatlas.org) were queried for data entries of marker identifiers. The *UrinePA* build contained high confidence peptide and protein identifications obtained from five labs using tandem MS proteomics [30]. About 2,500 non-redundant proteins were cataloged at 1% false discovery rate. Another database listed 587 entries of a “Core Urinary Proteome”, which was established from an in-depth analysis of second morning urine obtained over three days from seven healthy 25-35 year old volunteers [31].

### SRM assays

Ten tryptic surrogate peptides were first chosen for the protein markers based on *in silico* trypsin digestion and existing MS/MS data from our own lab, the Global Proteome Machine (GPM) and PeptideAtlas. These peptides were then evaluated by ESP predictor [32] and CONSeQuence [33] software. Three to five peptides with moderate hydrophobicity and high scores from the prediction tools were selected for peptide synthesis. The synthesized crude heavy-isotope labeled peptides were further evaluated in peptide response and fragmentation pattern. Optimal collision energy (CE) values were achieved by direct infusion of the individual peptides, and/or multiple LC-SRM runs with CE ramping. For each peptide, the three best transitions and matrix interference were determined. The relative intensity ratios among the three selected transitions for SRM were predefined by the internal standard heavy peptides in buffer. Matrix interference for a given transition that fell into mass widths Q1 and Q3 from co-eluting peptides was identified by a deviation from the expected relative intensity ratios among the transitions. The transition with no matrix interference was used for marker quantification in prostate urine samples. Before running the clinical cohort urine samples, the detectability of endogenous peptides in a pooled prostate cancer urine sample was systematically evaluated to finalize the best performing peptides for each protein marker. The detectable peptides were used for further quantification of the secreted protein markers in the cohort urine samples. For proteins with two or more detectable endogenous peptides, SRM signal correlation between any two surrogate peptides from the same protein was analyzed. For proteins with only one detectable endogenous peptide across all the urine samples, the potential of modifications on the surrogate peptides was evaluated by the knowledge-base information on PhosphoSitePlus and Uniport websites.

### LG-SRM

The LG-SRM approach was previously demonstrated in enabling reproducible quantification of target proteins at ~10 ng/mL levels in nondepleted human serum [16]. Typically, 4 μL of tryptic digest samples with a peptide concentration of 0.5 μg/μL were directly loaded onto a capillary reversed-phase column, 75 μm inner diameter (i.d.) × 150 cm length, packed in-house with 3-μm Jupiter C18 bonded particles (Phenomenex, Torrance, CA) to permit long gradient separation without a trap column with its dead volume affecting peptide retention time. Peptide separations were performed at a mobile phase flow rate of 100 nL/min on a binary pump system using 0.1% formic acid in water as phase A and 0.1% formic acid in 90% acetonitrile as phase B. The profile for a 300 min gradient time was 5–15% B in 27 min, 15–25% B in 140 min, 25–35% B in 73 min, and 35–90% B in 60 min. The TSQ Vantage mass spectrometer was operated in the manner as previously described [16].

### PRISM-SRM

The PRISM-SRM approach has been previously described for quantification of low-abundance proteins in human plasma or serum [17]. Briefly, high resolution reversed phase capillary LC with pH 10 mobile phase was used as the first dimensional separation of peptides from trypsin-digested human urine proteins. Following separation, the column eluent was automatically collected every minute into a 96-well plate during a ~100 min LC run while on-line SRM monitoring of heavy internal standard peptides was performed on a small split stream of the flow. Intelligent selection (termed *i*Selection) of target peptide fractions was achieved based on the on-line SRM signal of internal standard peptides. Prior to peptide fraction collection, 17 μL of water was added to each well to minimize excessive loss of peptides and to dilute the peptide fractions (~1:7) for LC-SRM analysis.

Following *i*Selection, the target peptide-containing fractions were subjected to LC-SRM measurement. All peptide fractions were analyzed by using the nanoACQUITY UPLC^®^ system (Waters Corporation, Milford, MA) coupled on-line to a TSQ Vantage triple quadrupole mass spectrometer (Thermo Scientific, San Jose, CA). Solvents used were 0.1% formic acid in water (mobile phase A) and 0.1% formic acid in 90% acetonitrile (mobile phase B). Peptide separations were performed at a mobile phase flow rate of 400 nL/min using an ACQUITY UPLC BEH 1.7 μm C18 column (75 μm i.d. × 10 cm), which was connected to a chemically etched 20 μm i.d. fused-silica emitter via a Valco stainless steel union. Four μL of individual peptide fractions (total volume 20 μL) following PRISM were injected for LC separations using a binary gradient of 10-20% phase B in 7 min, 20-25% phase B in 17 min, 25-40% phase B in 1.5 min, 40-95% phase B in 2.5 min and 95% phase B in 6 min for a total time of ~35 min. The TSQ Vantage was operated in the same manner as previously described [11, 17]. A scan width of 0.002 *m/z* and a dwell time of 40 ms were set for all SRM transitions.

### SRM data analysis

SRM data were analyzed using the Skyline software [34]. Peak detection and integration were determined based on (1) same retention time; (2) approximately same relative SRM peak intensity ratios across multiple transitions between light (L) peptides and heavy (H) peptide standards [11, 17, 35]. All data were manually inspected to ensure correct peak detection and accurate integration. Signal to noise ratio (S/N) was calculated by the peak apex intensity over the highest background noise in a retention time region of ±15 s for the target peptides [17, 35]. The background noise levels were conservatively estimated by visually inspecting chromatographic peak regions. Quantifiable endogenous surrogate peptides should have SRM signals with S/N ≥ 10. The RAW data from TSQ Vantage were loaded into Skyline to create high resolution figures of extracted ion chromatograms (XICs) of multiple transitions monitored for the target peptides = proteins.

### Statistical analysis

GraphPad Prism (v.6.0) was used for statistical analysis and plotting; *P* < 0.05 was considered statistically significant [11]. Receiver operating characteristic (ROC) curves were produced in terms of sensitivity and specificity of protein markers at their specific cutoff values to evaluate the diagnostic performance of each candidate biomarker. The optimal cutoff was the point with the best sum of sensitivity and specificity. Multivariate evaluative analysis for various combinations of protein markers was done using SPSS (v.16.0) by logistic regression to find the best-fitting model for each comparison group.

## SUPPLEMENTARY MATERIALS FIGURES AND TABLES























## Abbreviations

AGR2: anterior gradient 2
AUC: area under the receiver-operating characteristic curve
CE: collision energy
DRE: digital rectal exam
DTT: dithiothreitol
GPM: Global Proteome Machine
LC: liquid chromatography
LG: long gradient
L/H: the ratio of SRM signal from endogenous peptide over heavy-labeled internal standard
PRISM: high-pressure, high-resolution separation with intelligent selection and multiplexing
ROC: receiver operating characteristic
sPSA: serum PSA
SRM: selected reaction monitoring
TFA: trifluoroacetic acid
uPSA: urinary PSA
XIC: Extracted ion chromatogram
