# Supplementary material for: Multiplexed targeted mass spectrometry assays for prostate cancer-associated urinary proteins
Source: Oncotarget. 2017 Oct 9;8(60):101887–98. doi: 10.18632/oncotarget.21710 (PMC5731921; doi:10.18632/oncotarget.21710)
Supplement: Supplementary file 1 [file oncotarget-08-101887-s001.pdf]

Multiplexed targeted mass spectrometry assays for prostate cancer-associated urinary proteins

SUPPLEMENTARY MATERIALS

Supplementary methods

Secreted protein concentration calculation in urine (ng/100 µg of total urinary protein):

$[\text{Concentration}]_{\text{secreted protein}} = L/H \times IS\ (\text{fmol}/\mu L) \times MW \times 10^{-6}\ (\text{ng/fmol}) \times 200\ \mu L$

IS: internal standard

MW: protein molecular weight (g/mol)

100 µg of urinary protein: 200 µL of 0.5 µg/ µL urine peptide

Supplementary Table 1: The panel lists the query result of database *UrinePA*. The parameter "observed" could be used to indicate abundance.

See Supplementary File 1

Supplementary Table 2: 13 prostate cancer-associated secreted proteins and their surrogate peptides. For each surrogate peptide three best transitions without co-eluting interference were monitored.

See Supplementary File 2

Supplementary Table 3: Summary of SRM measurements of PSA protein in 27 clinical urine samples including 7 post-op subjects (two purified PSA internal standards IVGGWEC$_{amc}$EK and LSEPAELTDAVK were spiked at 1 fmol/µL and 10 fmol/ µL, respectively).

See Supplementary File 3

Supplementary Table 4: Estimation of the percentage of PSA from the post-op urine over PSA from the non-cancer urine (the surrogate peptide IVGGWEC$_{amc}$EK was used).

See Supplementary File 4

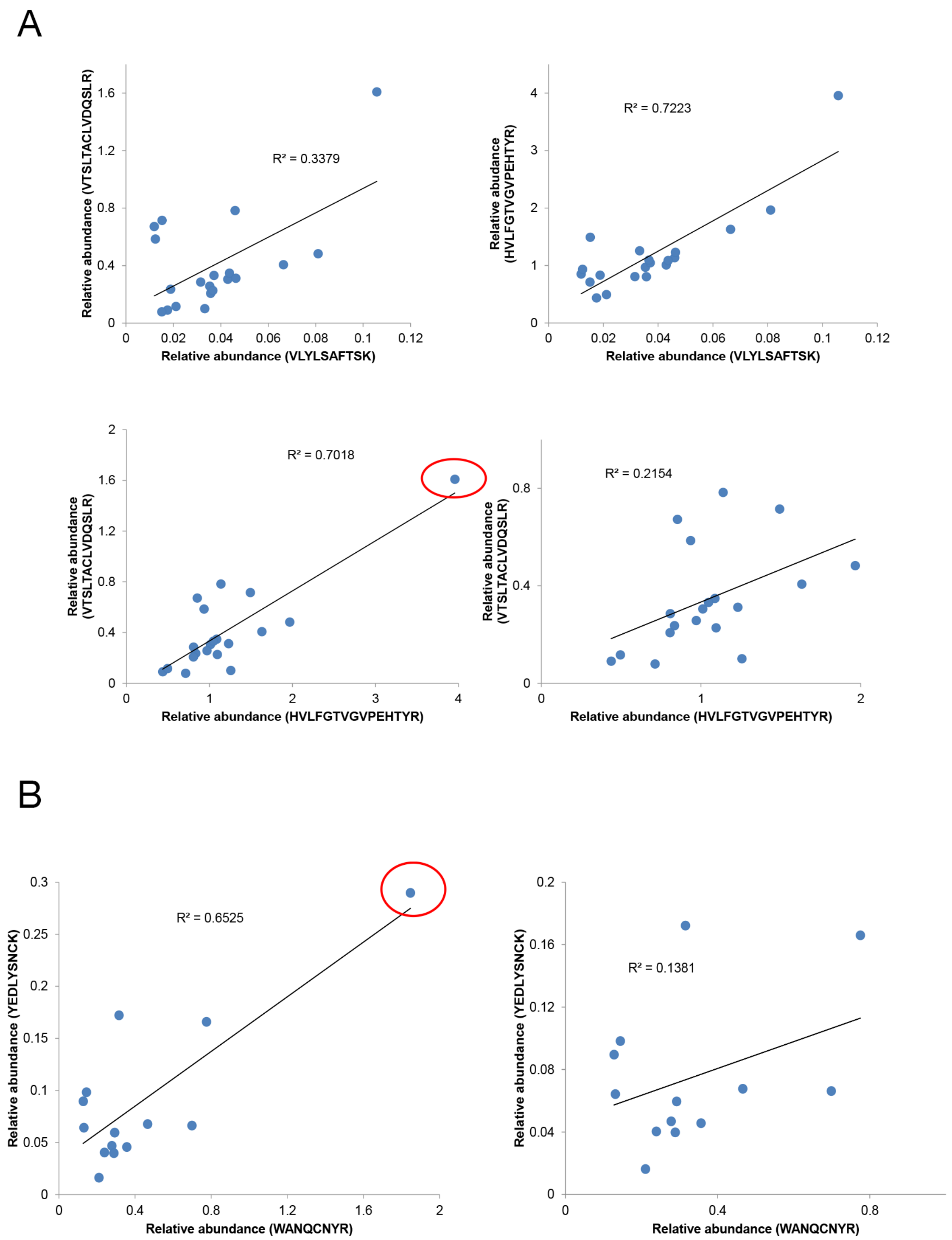

Supplementary Figure 1: Correlation curves between any two surrogate peptides from the same protein. **(A)** CD90 (with the removal of the point with the red circle, the correlation coefficient of R^2 was significantly dropped from 0.70 to 0.21); **(B)** CRISP3 (with the removal of the point with the red circle, the correlation coefficient of R^2 was significantly dropped from 0.65 to 0.14).

Gene Symbol	05-179_CD26t	08-032_CP_epi_CD26posi	Mean Intensity	Max Intensity	Gene Title
AGR2			2128.03	3943.90	anterior gradient 2 homolog (Xenopus ...
BCMP11			180.58	346.74	breast cancer membrane protein 11
CEACAM5			341.94	622.91	carcinoembryonic antigen-related cell...
CEACAM6			215.44	385.83	carcinoembryonic antigen-related cell...
CRISP3			1042.17	1844.03	Cysteine-rich secretory protein 3
IL24			1316.43	2147.29	interleukin 24
KLK3			9762.09	14749.51	Kallikrein-related peptidase 3
MMP9			1621.25	3004.10	matrix metallopeptidase 9 (gelatinase...

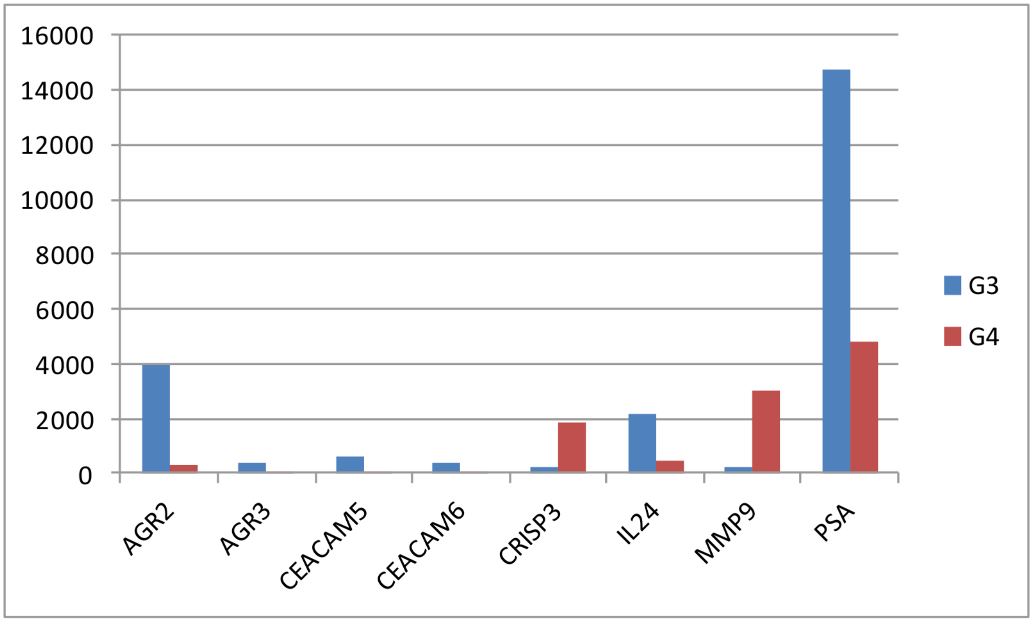

Supplementary Figure 2: Gene expression levels of protein markers in Gleason 3 (labeled 05-179_CD26t) *vs.* Gleason 4 (labeled 08-032_CP_epi_CD26posi) cancer cells. Differential expression is displayed on gray scale (top panel) and histogram (bottom panel) formats.

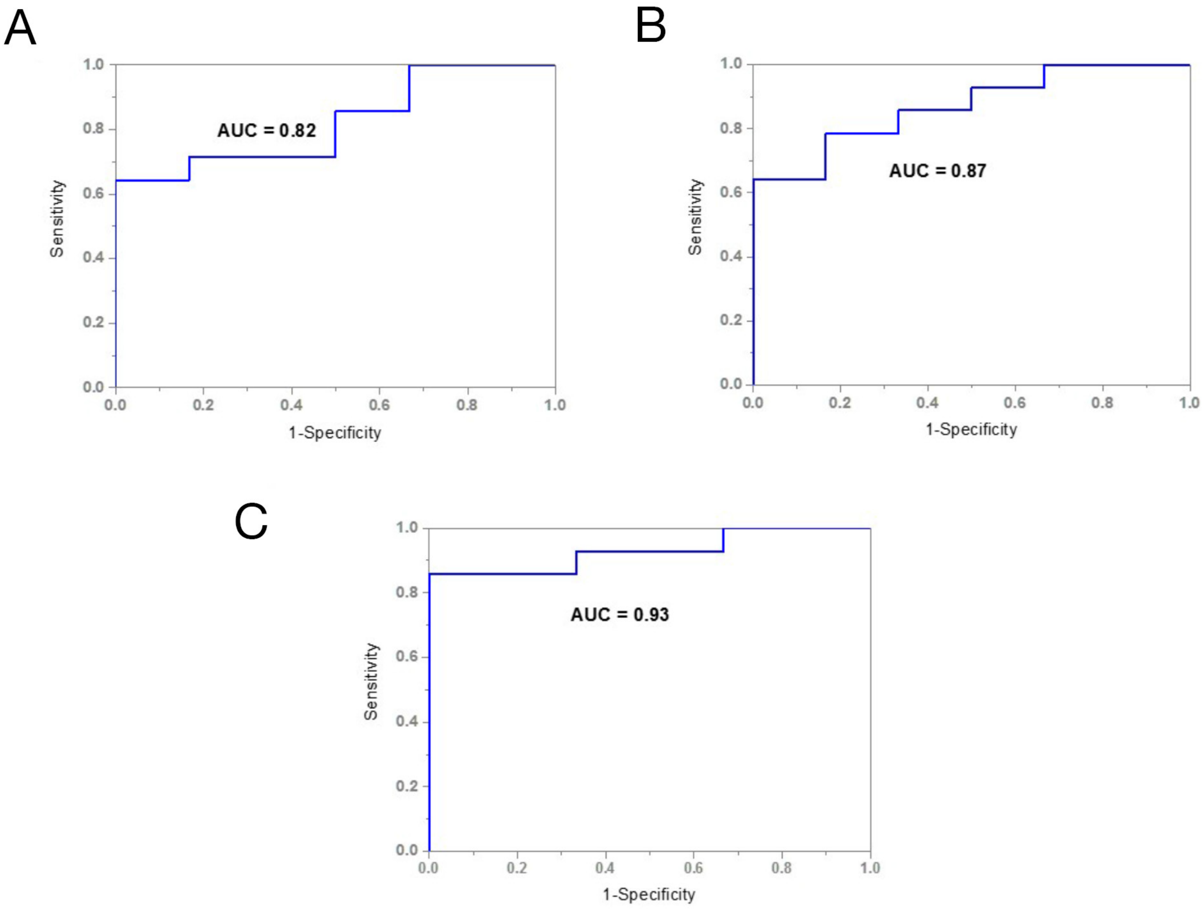

Supplementary Figure 3: ROC curve analysis for the combination of all surrogate peptides from the same protein. (A) CRISP3; **(B)** CD90; **(C)** MMP9.

Supplementary Table 5: Summary of multiplex SRM measurements of prostate cancer-associated secreted proteins in 20 clinical urine samples.

See Supplementary File 5

Supplementary Table 6: The SRM signal ratio of urinary secreted protein/PSA, i. e., $(L/H)_{\text{peptide marker}}/(L/H)_{PSA}$ from SRM measurements in 20 clinical urine samples (crude internal standards for prostate cancer-associated secreted proteins and purified internal standard for PSA surrogate peptide IVGGWEC$_{amc}$EK were spiked at 10 fmol/µL and 1 fmol/µL, respectively).

See Supplementary File 6

Supplementary Table 7: Selected combinations of multiple markers for achieving better discrimination than individual markers between cancer and non-cancer.

See Supplementary File 7

Supplementary Table 8: Combinations of all surrogate peptide markers from the same protein to evaluate whether there is an increased discrimination power when compared to individual peptide markers.

See Supplementary File 8

Supplementary Table 9: PSA concentrations in urine and serum for 20 measured subjects (urinary PSA and serum PSA concentrations were obtained from SRM measurements and ELISA measurements, respectively).

See Supplementary File 9

Supplementary Table 10: The ratios of secreted protein over PSA concentrations, urinary PSA (uPSA) and serum PSA between low volume/low grade cancer (n = 6) and significant cancer (n = 5). The low volume/low grade cancer: Gleason score ≤ 6 and tumor volume ≤ 0.5 cc; the significant cancer: Gleason score > 6 and tumor volume > 0.5 cc.

See Supplementary File 10
