# Supplementary figures and images for: Multiplexed targeted mass spectrometry assays for prostate cancer-associated urinary proteins
Source: Oncotarget. 2017 Oct 9;8(60):101887–98. doi: 10.18632/oncotarget.21710 (PMC5731921; doi:10.18632/oncotarget.21710)

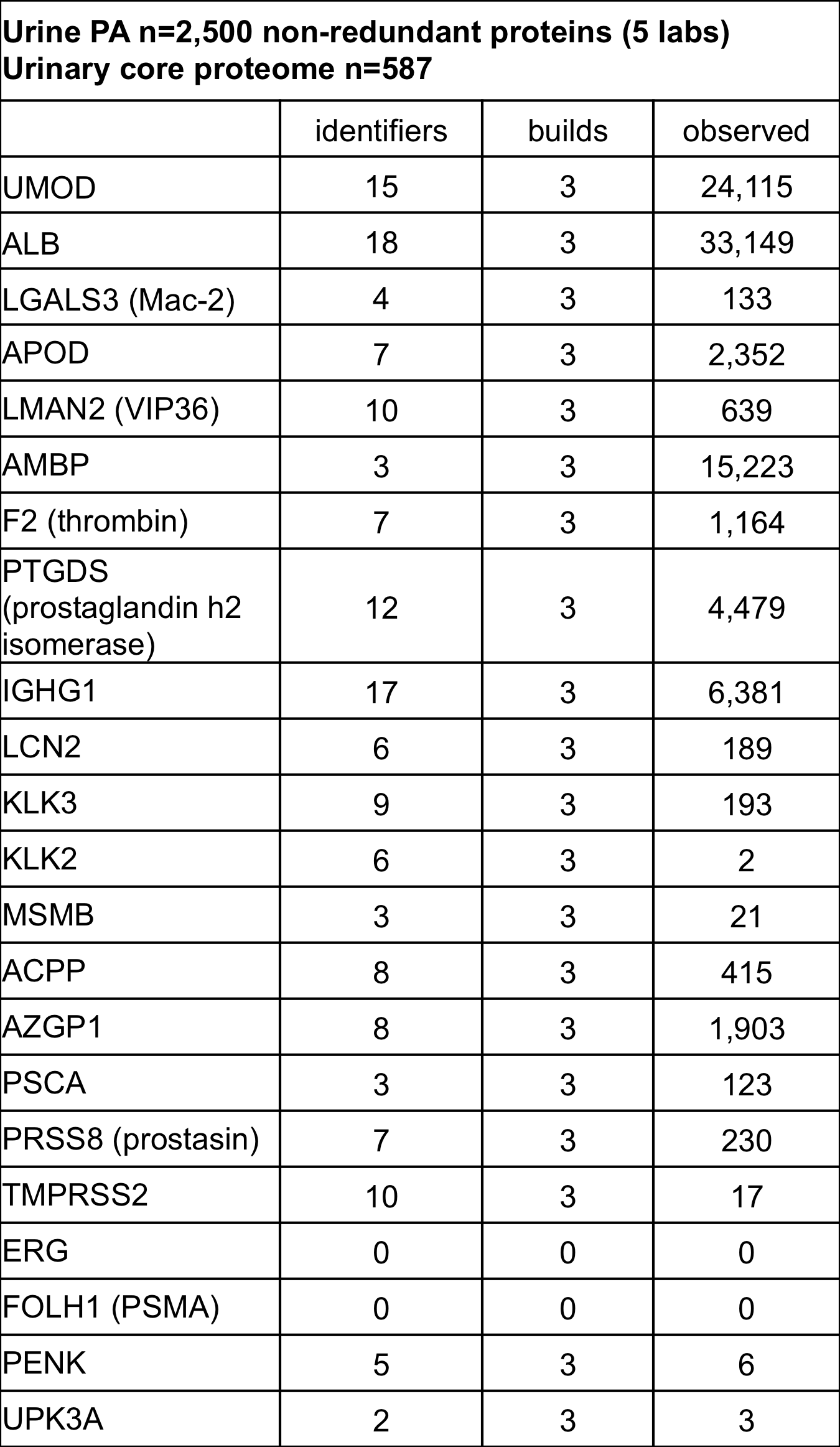

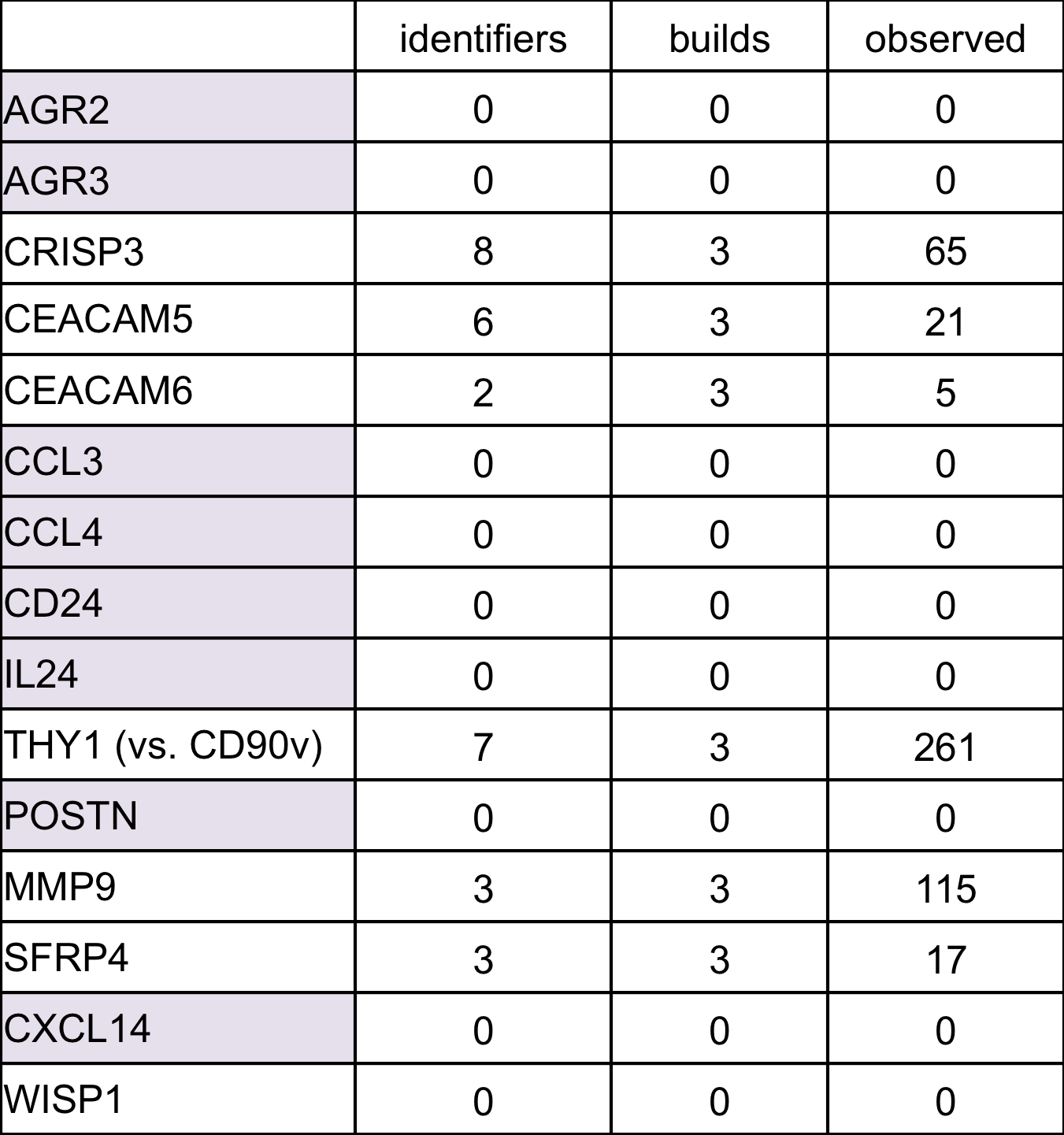

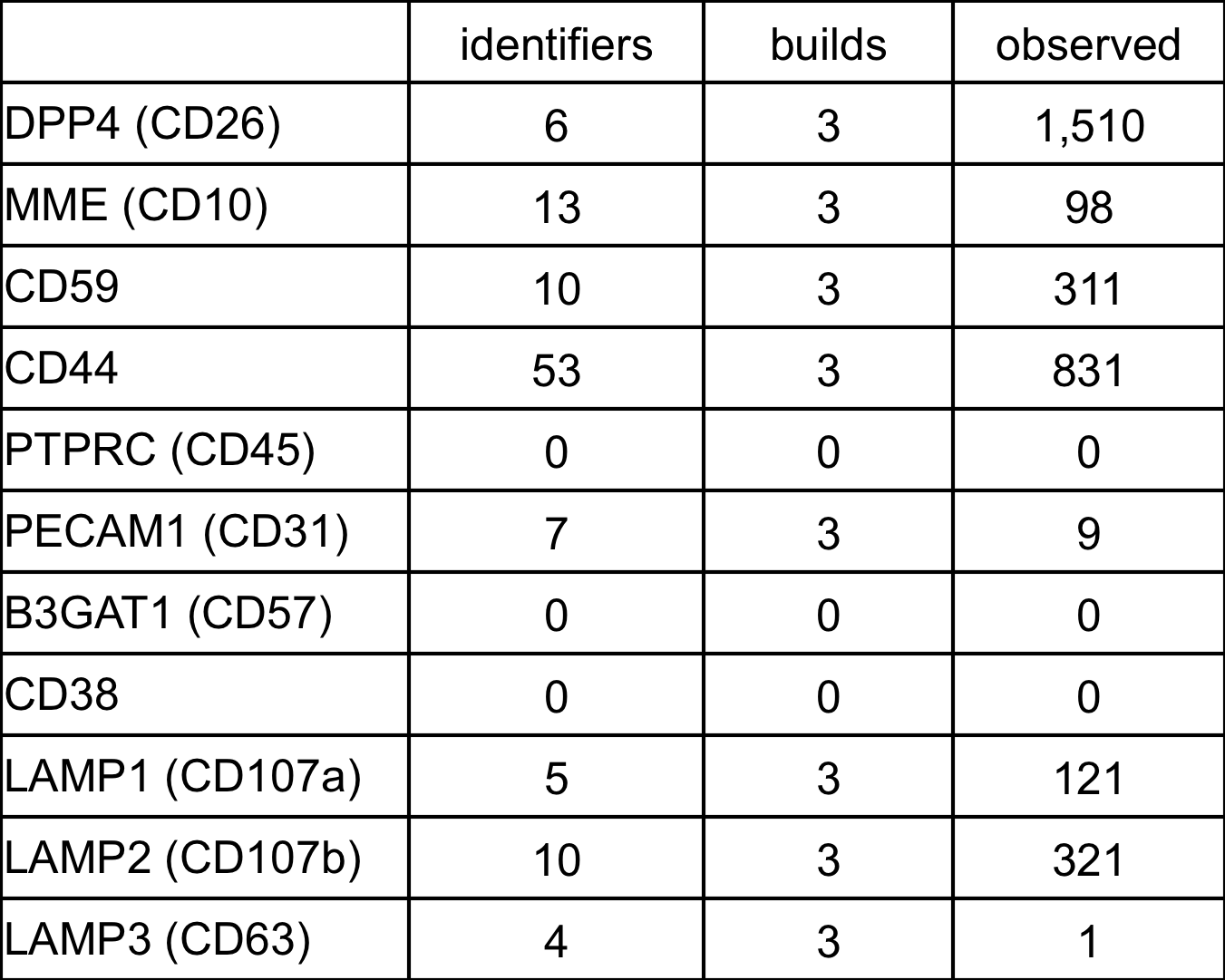


Supplementary Table 1

Supplement: Supplementary file 2 [file oncotarget-08-101887-s002.docx]

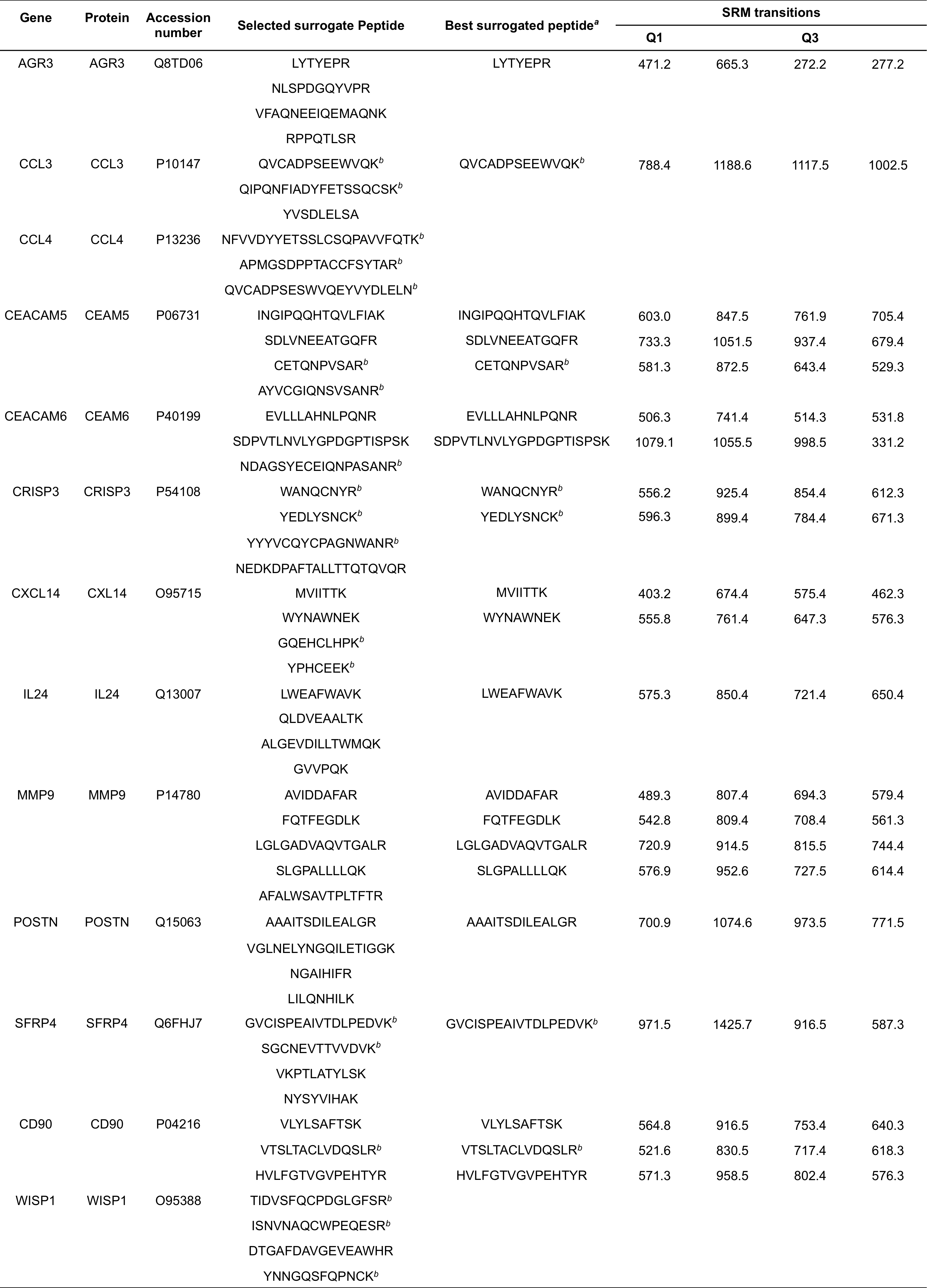


Supplementary Table 2

Supplement: Supplementary file 3 [file oncotarget-08-101887-s003.docx]

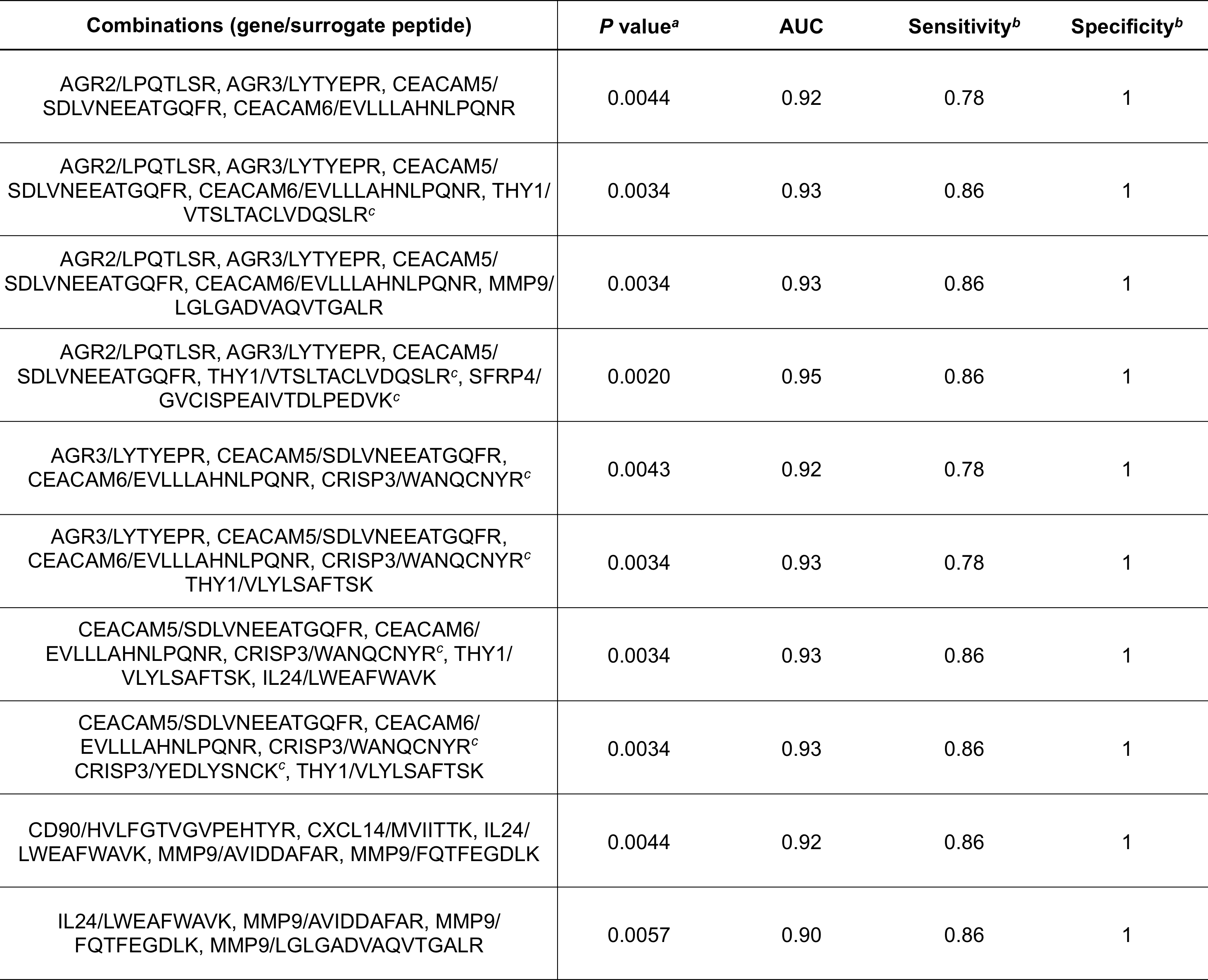


Supplementary Table 7

Supplement: Supplementary file 8 [file oncotarget-08-101887-s008.docx]

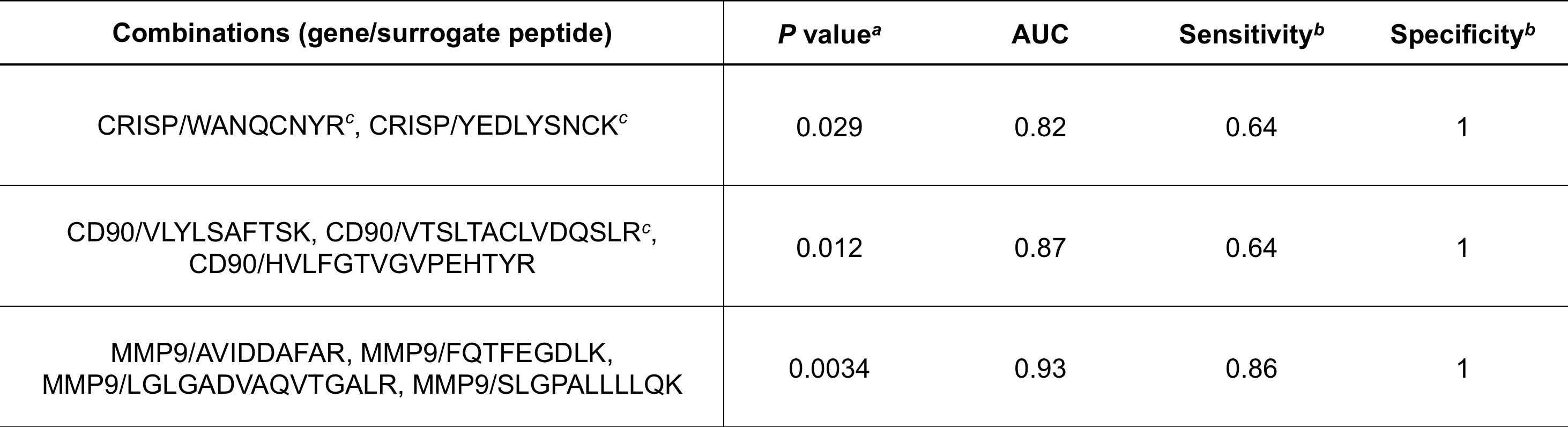


Supplementary Table 8

Supplement: Supplementary file 9 [file oncotarget-08-101887-s009.docx]
